# Developing the MyCancerGene Digital Health Portal to Improve Patients’ Understanding of Germline Cancer Genetic Test Results: Development, User, and Usability Testing Study

**DOI:** 10.2196/56282

**Published:** 2025-05-14

**Authors:** Phillip Trieu, Dominique Fetzer, Briana McLeod, Kathryn Schweickert, Lauren Gutstein, Brian Egleston, Susan Domchek, Linda Fleisher, Lynne Wagner, Kuang-Yi Wen, Cara Cacioppo, Jessica E Ebrahimzadeh, Dana Falcone, Claire Langer, Elisabeth Wood, Kelsey Karpink, Shelby Posen, Enida Selmani, Angela R Bradbury

**Affiliations:** 1 Division of Hematology-Oncology Abramson Cancer Center The University of Pennsylvania Philadelphia, PA United States; 2 Fox Chase Cancer Center Temple University Philadelphia, PA United States; 3 Grillings School of Global Public Health University of North Carolina Chapel Hill, NC United States; 4 Thomas Jefferson University Philadelphia, PA United States; 5 University of Pennsylvania Philadelphia, PA United States

**Keywords:** genetic health portal, digital intervention development, health portal, usability, digital health, germline cancer, multigene panels, clinical practice, likelihood, effectiveness, medical history, genetic medicine, risk information, digital tool, intervention, longitudinal care, patient-centered content, electronic information

## Abstract

**Background:**

The use of multigene panels has significantly increased the likelihood that genetic testing will leave patients with uncertainties regarding test interpretation, implications, and recommendations, which will change over time. Effective longitudinal care models are needed to provide patients with updated information and to obtain patient and family history updates.

**Objective:**

To bridge this gap, we aimed to develop a patient- and genetic provider–informed digital genetic health portal (GHP), MyCancerGene, to improve longitudinal patient understanding of and responses to genetic testing.

**Methods:**

We used a 5-step process to develop MyCancerGene. To better understand their interest in and willingness to use a digital GHP, we surveyed 307 patients who completed genetic testing (step 1). We completed qualitative interviews with 10 patients and a focus group with 17 genetic providers to inform the content and function of MyCancerGene (step 2). Next, we developed initial intervention content (step 3) and completed user testing of intervention content with 25 providers and 28 patients (step 4). After developing the prototype intervention, we completed usability testing with 8 patients for their feedback on the final content, functions, and ease of use (step 5).

**Results:**

In surveys conducted in step 1, 90% of patients with positive results reported interest in a digital GHP, and over 75% of participants with variants of uncertain significance or uninformative negative results reported similar interest. The most frequently reported advantages among patients were increasing accessibility, convenience, and efficiency (103/224, 46%); keeping genetic information organized (54/224, 24.1%); and increasing or maintaining patient understanding of the information (38/224, 17%). In qualitative interviews (step 2), both patients and genetic providers endorsed the benefit of the tool for updating personal and family history and for providers to share new risk information, test interpretation, or other medical changes. Patient and provider input informed eight key components of the tool: (1) Landing Page, (2) Summary of Care page, (3) My Genetic Test Results page, (4) My Family History page, (5) Provide an Update page, (6) Review an Update page, (7) Resources page, and (8) the Screenings Tracker. They also recommended key functions, including the ability to download and print materials and the inclusion of reminders and engagement functions. Potential challenges identified by patients included privacy and security concerns (67/206, 32.5%) and the potential for electronic information to generate distress (20/206, 9.7%). While patients were comfortable with updates (ie, even variant reclassification upgrades or clinically significant results), 44% (11/25) of genetic providers were uncomfortable sharing variant reclassification upgrades through MyCancerGene.

**Conclusions:**

MyCancerGene, a patient-centered digital GHP, was developed with extensive patient and genetic provider feedback and designed to enhance longitudinal patient understanding of and affective and behavioral responses to genetic testing, particularly in the era of evolving evidence and risk information.

## Introduction

### Background

Advances in basic science in genetics have shown great promise in improving human health and reducing the burden of cancer [1,2]. The promise of precision medicine is the ability to tailor treatment or screening of individual patients based on their genotype. With these advances, there is an increasing need for multidisciplinary translational research that focuses on how to advance precision medicine discoveries into clinical practice and to capitalize on connectivity (eg, digital health solutions) in ways that benefit the health of entire populations [3-5].

One of the challenges with recent advances in clinical sequencing, including hereditary cancer multigene panel testing (MGPT), is the increasing likelihood that genetic testing will leave many patients with uncertainties regarding interpretation, implications, and recommendations, all of which will change over time. Many of the genes included in multigene panels are moderate-penetrance genes, increasing the risk of cancer by only 2- to 4-fold, and in many cases, risk estimates are based on limited data and continue to evolve over time [6-8]. There is also uncertainty regarding optimal screening, given medical management recommendations, such as those published by the National Comprehensive Cancer Network and others, are continually evolving as new data emerge. Thus, even positive results leave many unknowns regarding cancer risks, optimal management, and the value of testing unaffected relatives [6,9,10]. In addition, multigene panels have been associated with higher rates of variants of uncertain significance (VUS), which are difficult for patients and genetic providers to understand, and can be reclassified over time [11-15].

This clinical transition from discrete (ie, single gene) to broad (ie, multi, whole genome) sequencing in clinical genetic testing presents challenges that will only increase as precision medicine applications expand. With broader testing, an increasing number of patients will be left at a risk of misunderstanding, uncertainty, and evolving interpretations and recommendations [8,16]. While these uncertainties will be clarified over time, we lack clinical care models to maintain longitudinal communication with patients to provide updated information regarding their genetic test result or medical recommendations and to obtain personal and medical history updates [16-19]. For example, in the current standard of care, if a genetic variant interpretation is updated (eg, VUS to benign or VUS to likely pathogenic result), the commercial laboratory contacts the ordering genetic provider, who in turn contacts the patient [20,21]. This model is not sustainable and may not be feasible due to outdated patient contact information or change in employment of providers. Alternatively, patients can contact their provider periodically, but this places a burden on patients and has not been proven to be successful.

Equally important, while many studies have shown limited psychosocial distress with genetic testing, robust longitudinal data on cognitive, affective, and behavioral outcomes in diverse and representative patient populations and with MGPT are limited [22,23]. Recent studies suggest some subgroups are at risk for greater posttest distress with MGPT, including patients with positive results, a history of cancer, and lower formal education [24-26]. Patients with positive and VUS results have also demonstrated greater distress and uncertainty [15,27,28]. Furthermore, there is an increased use of remote counseling (eg, phone or videoconference), digital alternatives, and streamlined counseling models, which may introduce additional short-term or longitudinal knowledge gaps or testing-related distress [29-33]. Thus, effective, evidence-based alternative strategies for longitudinal communication and care for genetic patients are critical to realizing the promise of precision medicine.

The Institute of Medicine has highlighted the importance of patient-centered care and improved transparency to enhance the delivery and outcomes of medical care [34-36]. Data from the Pew Research Center in 2021 report that 93% of Americans use the internet, 80% have searched the web for health information, and patients frequently have high interest in communicating electronically with genetic providers [37-41]. Thus, interactive health communication applications, also known as digital health solutions or tools (eg, apps, health care portals, and educational or decision aid interventions), have been implemented to facilitate this goal of enhanced transparency and communication [36,42-44]. Digital health tools may be particularly effective in chronic disease management, where longitudinal care is crucial, when informational needs change over time or vary among patients, when coping and adjustment are part of the ‘journey,’ and when improved medical outcomes require changing health behaviors [43]. Studies from a variety of chronic disease settings, excluding genetics, have shown that digital health tools can improve knowledge [43], self-efficacy [43,45,46], satisfaction [45,47], clinical outcomes [43,47-50], and unmet communication needs [45,48]. Recent randomized studies have shown reductions in distress with digital health tools [46,49,51]. Furthermore, digital health tools may provide education in a way that is more simple, accessible, and salient, resulting in better knowledge retention [43].

Yet, many studies have identified limitations and knowledge gaps related to digital health tools [36,42,44,45,52,53]. Various types of digital health tools have been used, and electronic patient portals with limited functions (ie, providing access to records only) may have less impact on outcomes [36]. Most randomized studies involving health portals have been relatively small or report limited outcomes or benefits [37,45,52]. Furthermore, in some studies, participants rarely or never log on to the portal [50], which may impact outcomes and diminish power and has been associated with factors such as race, ethnicity, and level of education [50,54-58]. In addition, studies including socioeconomically diverse patient populations are needed, as minority patients are significantly less likely to use electronic patient portals, which could exacerbate health care disparities [37,45,54,59-62]. Importantly, at the time we developed MyCancerGene, there were no published studies evaluating longitudinal digital health tools in clinical cancer genetics, which shares many of the characteristics of chronic illness (eg, evolving information over time, adjustment and need for behavior change, and communication with relatives and other health care providers). Thus, we propose that cancer genetic testing is an ideal clinical context to study the benefits and limitations of a theoretically (ie, diffusion of innovation theory) and patient- and genetic provider–informed patient-centered longitudinal digital health tool to optimize patients’ outcomes following cancer genetic testing and the clinical implementation of precision medicine.

### Objective

To address this clinically substantial gap in the delivery of genetic medicine, we sought to obtain patient and genetic provider input to develop a patient- and provider-informed longitudinal digital genetic health portal (GHP) called MyCancerGene to enhance longitudinal patient understanding of and affective and behavioral responses to genetic testing, particularly in the era of evolving evidence and risk information.

## Methods

### Overview

We used a 5-step process to develop the MyCancerGene digital tool (Table 1). First, we describe initial patient and genetic provider feedback and recommendations regarding the concept of MyCancerGene. Next, we describe user and usability testing, which informed the final content and functionality of MyCancerGene.

**Table 1:** Summary of the 5 steps, methods, and outputs involved in the development of MyCancerGene. Each step included feedback and input from both patients and genetic providers (N=395).

Steps	Methods	Outputs
1. Inquire	Surveyed patients (n=307) who have completed genetic testing in the multicenter COGENT^a^ study	Evaluated participants’ interest in and barriers to a digital health portal Explored GHP^b^ advantages, disadvantages, and usefulness and potential content
2. Determine	Individual qualitative interviews with patients (n=10) and a genetic provider focus group (n=17) to better understand key intervention components and functions	Informed by the diffusion of innovation theory, evaluating key attributes (eg, relative advantage, risk compatibility, and complexity) Evaluated patient and provider preferences for content and functionality, including comfort with updates in test results
3. Develop	Incorporate feedback into MyCancerGene	GHP (MyCancerGene) informed by formative interviews Key components from patient and provider input Initial screenshots were developed for user testing
4. User testing and refinement of the intervention	Provider (n=25) and patient (n=28) feedback on the specific drafted content and functionality	Feedback on the purpose, content, and comfort with specific functions Recommendations for changes
5. Usability testing and final modifications to the intervention	Patient feedback (n=8) on the initial digital version of the intervention	Feedback on content, presentation, and functionality of the initial digital intervention

^a^COGENT: Communication of Genetic Test Results by Telephone.

^b^GHP: genetic health portal.

### Step 1: Evaluating Patient Interest in a Digital GHP

To better understand interest in and willingness to use a digital GHP among patients who had undergone clinical cancer genetic testing, we surveyed patients in the National Institutes of Health–funded Communication of Genetic Test Results by Telephone (COGENT) study. The COGENT study was a multicenter, noninferiority randomized study of telephone disclosure compared to in-person disclosure of cancer genetic test results, including MGPT [29,63]. All participants in the COGENT study were English-speaking adults who had completed in-person pretest counseling with a genetic counselor and were proceeding with clinical cancer genetic testing. As participants in the study, they completed surveys before and after the disclosure of results. This was an ideal clinically diverse population to obtain patient feedback on the value, advantages, disadvantages, and content for the development of a future digital GHP (eg, MyCancerGene).

To obtain patient feedback, we develop closed- and open-ended items that were added to the COGENT postdisclosure surveys in 2014. These included 7 close-ended questions about the interest in and barriers to a GHP, as well as 9 open-ended items asking how a GHP could be helpful, its advantages and disadvantages, and what types of information and functions would be most useful. Framework analysis was used to examine open-ended responses [64-66]. Investigators reviewed responses for a subsample (58/307, 18.9%) of participants and developed a thematic framework of primary and secondary themes for each open-ended item. Next, 2 investigators (PT and AB) independently assigned thematic codes to the open-ended responses and discussed differences to refine the thematic framework. The thematic framework was then applied to the remaining samples’ open-ended responses, and themes were refined to include new ones as they emerged. Differences in code assignments were resolved through discussion and establishing agreement for all responses.

### Step 2: Qualitative Patient and Genetic Provider Inquiry to Inform the MyCancerGene Intervention

To better inform specific content and functions of our initial GHP, we conducted additional qualitative interviews with patients and genetic providers. Interviews were guided by the Diffusion of Innovation Theory and key attributes of successful innovations (eg, advantage, risk, compatibility, and complexity) were evaluated [67-70]. Genetic providers included 13 genetic counselors and 4 physicians with expertise in cancer genetics (n=17) at the 5 COGENT sites, who participated in a provider focus group in 2014. All providers were female and had practiced in cancer genetics for many years. Providers were asked open-ended items evaluating the perceived usefulness of a GHP for patients and providers, perceived provider challenges, and comfort with different types of updates (eg, reminders, general testing updates, upgraded and downgraded VUS results, and patient family history updates). Open-ended responses were independently coded as described earlier by 2 authors (PT and KS) and resolved through discussion with a third coder (BM).

Patients included 10 purposefully selected COGENT participants across sites to represent the view of sociodemographically diverse patients who had undergone genetic testing. Patients completed individual interviews, including open-ended questions in 2014. These included (1) evaluating internet and health portal use in general, (2) advantages and disadvantages to health portals, (3) advantage and disadvantages to a GHP, (4) content that would be useful and not useful, (5) perceived usefulness of updates in genetic information and updates in family history, and (6) other suggestions for the GHP content and function. Similarly, the open-ended responses as described earlier were independently coded by 2 authors (PT and KS) and resolved through discussion with a third coder (BM).

### Step 3: Developing the MyCancerGene Intervention

Key components and functions of our GHP (MyCancerGene) were informed by our formative interviews of the patients and genetic providers. Initial screenshots were developed for patient and provider feedback. Genetic counselors involved in content development reflected on their clinical experiences, providing clinical counseling to patients of diverse backgrounds during intervention content development. Similarly, researchers with expertise in health disparities and behavioral science were attentive to development for a clinically diverse population. Strategies included using plain language, definitions, short sentences, and more inclusive images.

### Step 4: User Testing and Refinement of the Intervention

Initial screenshots for MyCancerGene content were developed based on the formative interviews of the patients and genetic providers, and individual user–testing feedback on these screenshots was obtained from both patients and providers in this step.

We obtained feedback from patients at 2 time points. The first group of patients providing feedback on user testing included 8 participants in 2014, and the second included 20 participants in 2019. In both sets of interviews, we purposefully selected for sociodemographically diverse participants. They viewed current versions of screenshots and were asked if the content was useful, what they expected to see, and why or why not for each question. The screenshots were updated in 2019 based on the feedback from 2014, although the questions were the same. In addition, they were asked for additional recommendations to improve the content. Participant responses were transcribed and summarized to inform modifications for subsequent interviews and development.

We conducted individual user–testing interviews with 25 genetic counselors from 2018 to 2019. Interviews included questions about the use of patient portals in general (ie, 7 items); feedback on the purpose, content, and comfort with specific functions of MyCancerGene (ie, 7 items); and feedback on individual screenshots and draft messaging for updates (ie, 14 items). Example questions are included in the associated tables. Participant responses were transcribed and summarized independently by 2 research staff to identify potential modifications. Feedback from the patients and providers were incorporated into the initial digital version of the MyCancerGene intervention.

### Step 5: Usability Testing and Final Modifications to the Intervention

Using the initial digital version of MyCancerGene and cobrowsing software, we conducted individual usability interviews with 8 purposefully selected patients to obtain feedback on the functionality, navigation, experience, and any additional comments on the content from 2020 to 2021. Participants were asked what they liked and disliked on each page, if the information was understandable, and how it could be clarified if not. As applicable, they were asked about fonts, colors and images, how easy it was to navigate the functions, and how the content or functions could be improved. Participant responses were transcribed and summarized independently by 2 research staff to identify potential modifications.

### Ethical Considerations

This study protocol was approved by the institutional review board (832,628), as well as the Cancer Center’s Clinical Trials Scientific Review and Monitory Committee at the University of Pennsylvania and was determined to pose minimal risks to participants All participants provided a signed or verbal (ie, depending on step) informed consent. All data were deidentified before analysis. Participants were compensated in a range from US $0 to US $25, depending upon stakeholder group and step. None of the participants are identifiable in any of the findings reported in this manuscript.

## Results

### Step 1: Evaluating Patient Interest in a Digital GHP

In total, 307 COGENT participants completed the self-administered GHP patient items as presented in Table 2.

Most patients reported a GHP would be useful and that they would use it as presented in Table 3. Those with a positive result were significantly more likely to report that a GHP would be helpful (odds ratio [OR] 10.9, 95% CI 2.2-54.0, *P*=.003) and that they would use a GHP (compared to true negatives: OR 7.2, 95% CI 2.1-25.0, *P*=.002). Nonetheless, over 75% (25/33) of those with a VUS or uninformative negative results reported a GHP would be helpful. Another factor associated with likely GHP use was history of prior health care portal use (OR 2.5, 95% CI 1.4-4.4, *P*=.001). Age, gender, education, cancer history, and baseline cognitive and affective measures (eg, knowledge and anxiety) were not substantially associated with interest in a GHP or the likelihood of using it.

**Table 2:** Characteristics of patients from the COGENT^a^ study who completed genetic testing and were asked about their interest in a potential genetic health portal (N=307).

Characteristics		Values
Age (y), mean (SD)		47.60 (12.86)
**Sex, n (%)**		
	Female	286 (93.2)
	Male	21 (6.8)
**Education, n (%)**		
	Some high school or less	1 (0.3)
	High school	15 (4.9)
	Associate degree, some college, or trade school	79 (25.7)
	College	107 (34.9)
	Post college	104 (33.9)
	Refused to answer	1 (0.3)
**Race, n (%)**		
	Asian	5 (1.6)
	Black or African American	16 (5.2)
	Mixed	4 (1.3)
	White	282 (91.9)
**Ethnicity, n (%)**		
	Hispanic or Latino	4 (1.3)
	Non-Hispanic or Latino	303 (98.7)
Married, n (%)		226 (73.6)
**Disclosure method, n (%)**		
	In person^b^	173 (56.4)
	Telephone	134 (43.6)
**Site**, **n (%)**		
	University of Pennsylvania	105 (34.2)
	Fox Chase Cancer Center	110 (35.8)
	University of Chicago	50 (16.3)
	Stroger Hospital at Cook County	7 (2.3)
	MD Anderson Cancer Center at Cooper	35 (11.4)
Known mutation in the family, n (%)		74 (24.1)
History of cancer, n (%)		153 (49.8)
**Result, n (%)**		
	Positive	52 (16.9)
	True negative	32 (10.4)
	Uninformative negative	189 (61.6)
	Variant of uncertain significance	34 (11.1)

^a^COGENT: Communication of Genetic Test Results by Telephone.

^b^In total, 53 patients were in the self-selected in-person group.

**Table 3:** Patient interest in a genetic health portal by genetic test results among patients who had completed genetic testing in the COGENT^a^ study (N=307).

	All, n (%)	Positive (n=50-52)^b^, n (%)	Uninformative negative (n=179-188)^b^, n (%)	True negative (n=30-32)^b^, n (%)	VUS^c^ (n=32-34)^b^, n (%)
Would a genetic health portal be helpful?^d^ (n=303)	249 (82.2)	48 (96)	154 (81.9)	22 (68.8)	25 (75.8)
Any barriers to using a genetic health portal?^e^ (n=291)	60 (20.6)	13 (26)	36 (20.1)	6 (20)	5 (15.6)
Would you use a genetic health portal?^f^ (n=305)	227^g^ (74.4)	48^g^ (92.3)	135^g^ (72.2)	20^g^ (62.5)	24^g^ (70.6)

^a^COGENT: Communication of Genetic Test Results by Telephone.

^b^A range is included as some participants did not answer all items.

^c^VUS: variant of uncertain significance.

^d^“Now that you have received your results, do you think it would be helpful to have access to a secure, password protected electronic Genetic Health Portal (similar to other health portals you might have used in the past) with the information you received from your genetic provider (genetic counselor, physician, nurse practitioner, physician assistant)? (Yes/No).”

^e^“Do you think there would be any challenges with and/or barriers to using a Genetic Health Portal?”

^f“^How likely is it that you would use a Genetic Health Portal?”

^g^Indicates somewhat or very likely on a 5-point Likert scale.

Table 4 presents patient feedback on why a GHP might be considered helpful, based on responses from 224 participants. The reasons were categorized into themes, highlighting aspects such as accessibility, organization, and the emotional benefits of having secure, easy access to genetic information. Conversely, some patients expressed concerns about the need for more human interaction, technology challenges, and potential privacy issues, which could make the portal less helpful for them. In response to open-ended questions presented in Table 4, the most frequently reported advantages of the GHP were increasing accessibility, convenience, and efficiency (103/224, 46%), keeping genetic information organized (54/224, 24.1%), and to increase or maintain patient understanding of the information (38/224, 17%). Patients also reported it could be helpful for sharing genetic information with others, downloading and printing documents, security, and reducing anxiety.

Table 5 summarizes the perceived disadvantages of using a GHP, based on feedback from 206 participants. The most common concerns include issues related to privacy and data security, potential emotional distress, and difficulties with technology, such as recalling log-in information or dealing with technical glitches. Some participants also noted that they might not use the portal frequently or prefer human interaction over digital communication. A subset of patients (35/224, 15.6%) reported that a GHP would not be helpful (Table 4), citing the lack of human interaction, potential access challenges and concerns regarding privacy, and the potential to cause anxiety. When asked specifically about potential disadvantages to a GHP, 36.4% (75/206) of participants reported no disadvantages and 32.5% (67/206) reported concerns about privacy. Other disadvantages reported at lower frequencies included that information could be distressing, trouble remembering log-in information, technical challenges, and the lack of human interaction as shown in Table 5.

Table 6 outlines the types of information that 227 participants indicated would be most useful in a GHP. The most frequently reported components or functions included access to results (112/227, 49.3%), medical recommendations (47/227, 20.7%), and an easy-to-understand review of results (29/227, 12.8%). Other suggestions included access to genetic providers and additional resources, appointment details, screening results, and family history (Table 6). Some participants also endorsed the value of a summary page, updates in information, and the ability to print and share materials with relatives or providers.

**Table 4:** Reasons a genetic health portal would be helpful^a^ (N=224).

Themes			Examples		Participants, n (%)
**Reasons a genetic health portal would be helpful**					
	Accessibility (85 participants mentioned convenience or accessibility, including access at any time; 18 participants mentioned efficiency, ie, being fast or quick)	“So that the information is readily accessible.” “Again, it’s just more convenient to have access to information whenever I choose.” “Because I would be able to refer back to the results. It would also be good to be able to see anything that I might have missed.”		103 (46)	
	Organization	“Paper records can be misplaced and this is a way to access the information.” “I would like to be able to electronically see my results so I don’t have to keep track of the printout.” “Having access to electronic records alleviates having to keep track of paper records.”		54 (24.1)	
	Maintaining understanding	“A Genetic Health Portal would be a central information resource for information related to my specific results and general genetic information. A portal could provide a central source for information and resources.” “Access to information is always helpful, especially when details are important and the information is sensitive.”		38 (17)	
	Ability to share results with others (6 participants specifically referred to the ability to download and print results)	No examples		26 (11.6)	
	Helpful (not otherwise specified)	No examples		13 (5.8)	
	For security (benefit of keeping results secure)	“No one else should have access to my genetic information.” “[This] would be a safe way to communicate with genetic provider.”		7 (3.1)	
	Emotional benefit	“I feel like it would empower me more to be in control of my own health information for my future.”		7 (3.1)	
	Ability to update information	No examples		1 (0.4)	
**Reasons a genetic health portal may not be helpful**					
	Lacks human interaction	“I think it would be worrisome to see this information without the assistance of someone knowledgeable explaining it to me... The genetic information is so sensitive and in some cases, unclear as to its significance regarding the individual and family members and really requires assistance to understand it.”		14 (6.3)	
	Not helpful if you lack access to or comfort with technology	“However, those that are not internet savvy will have difficulties.” “I haven’t used (or care to use) health portals because, quite frankly, creating accounts/passwords for so many varied things has become too frustrating for me personally).”		9 (4)	
	No benefit or advantage	“I prefer meeting in person so I can receive a thorough explanation and have the opportunity to ask questions.”		7 (3.1)	
	Concern for security breach or privacy	“Might be nice. Concerned about security of personal information.”		7 (3.1)	
	Could cause anxiety	“But I always worry about finding bad news this way [through GHP] and then having to wait to see doctor.”		5 (2.2)	
	Not helpful because not combined with other portals	No examples		1 (0.4)	

^a^“Now that you have received your results, do you think it would be helpful to have access to a secure, password protected electronic Genetic Health Portal (similar to other health portals you might have used in the past) with the information you received from your genetic provider (genetic counselor, physician, nurse practitioner, physician assistant)? Why or Why not [coded responses].”

**Table 5:** Disadvantages of a genetic health portal^a^ (N=206).

Theme	Examples	Participants, n (%)
None	No examples	75 (36.4)
Not secure enough and concerns regarding privacy (risk of breach)	“Data could be hacked and used against for insurance or employment purposes.” “The only disadvantage would be if the site was breached.” “I suppose digital security is always a concern.”	67 (32.5)
Upsetting or distressing	“Genetic testing that could prove to be more anxiety-provoking than beneficial.” “Finding out some life changing news without the comfort or clarification from a person.” “For some, upsetting way to get bad news, perhaps misunderstanding information.”	20 (9.7)
Difficulty recalling log-in information or too many health care sites	“One more password to remember/forget.”	12 (5.8)
Technical challenges (6 participants mentioned that it would be hard for those who lack technical skills, 2 mentioned that some may lack access to technology, and 2 mentioned glitches, website malfunctioning, and maintenance)	“It would not be so beneficial for people who do not have access to online portals or do not know how to use the system.” “If I didn’t have a computer and had no access to a computer.” “Any possible glitches with the system.”	10 (4.9)
May not use (too much time or no benefit)	“Not referred to often enough.” “The time to sit down and look up/use the portal.”	7 (3.4)
Lack of human interaction	“Less personal than talking to someone.”	5 (2.4)

^a^“What do you feel would be the disadvantages to having access to the Genetic Health Portal?”

**Table 6:** Types of information desired for a genetic health portal^a^ (N=227).

Theme	Participants, n (%)
Genetic test results	112 (49.3)
Educational or informational resources (26 participants mentioned resources and education; 20 statistics, cancer risks, or other numerical information; 19 current research or updates in research; and 2 the results of this research)	62 (27.3)
Medical recommendations	47 (20.7)
Easy-to-understand explanation of results	29 (12.8)
Genetic provider information and notes (14 participants mentioned access to clinicians or contact information, 7 provider notes, and 3 medication details)	24 (10.6)
Appointment information (18 participants mentioned appointment details, such as dates seen, who did I see, or location of visit; 6 upcoming appointments or reminders for upcoming appointments)	22 (9.7)
Everything	18 (7.9)
Results of screening or procedures	16 (7.1)
Family history	14 (6.2)
Genetic test description	14 (6.2)
Summary (quick overview or snapshot)	10 (4.4)
Updates to recommendations, new information, or research	9 (4)
Print and share function (to share with relatives or providers)	6 (2.6)
Ways to share or engage with others (other patient experience or patient forum)	3 (1.3)
Recommendations for relatives	1 (0.4)
Billing or insurance	1 (0.4)

^a^“What type of information or documents do you think would be most useful to include in the Genetic Health Portal?”

### Step 2: Qualitative Patient and Genetic Provider Inquiry to Inform the MyCancerGene Intervention

#### Qualitative Genetic Provider Focus Group

The primary potential benefits of a GHP, as reported by genetic providers, included mechanisms to improve patients’ sharing of accurate information with relatives and providers and for providers to update patients with new risk or test information. Most providers felt a GHP would be useful to patients as they already expect providers to update them (ie, “call them”) with new information, although providers admitted this is not always feasible in practice and over time. Many endorsed a GHP as a way for patients to maintain their genetic records electronically in a specific location instead of in paper files. At the same time, providers indicated that many patients already have electronic access to these records through the electronic health record (EHR) and asked how the GHP would be different from the access already provided.

Providers acknowledged that they were not sure that a GHP would alleviate their day-to-day challenges as they already communicate with some patients via the EHR. Other concerns with a GHP included ensuring that patients receive and understand updates shared through the GHP, and that many patients do not log on or use ancillary platforms. Genetic providers asked if there could be an alert to providers if their patient logged on and reminders to patients when new information became available. Other concerns were regarding the burden on providers to update a GHP, maintaining current information, and the potential to create additional work for providers.

Genetic providers had different levels of comfort with updates provided through a GHP. They all felt that a GHP could be a good method to alert patients to update their family history (eg, suggested once or twice a year). They were less comfortable with updates to screening recommendations as these could change overtime and could conflict with other information or recommendations patients’ were receiving. In general, most providers were comfortable with new general information about testing (eg, new testing available) and downgraded VUS reclassifications. At the time, VUS downgrades were communicated via letter and a GHP could provide a good alternative to mail communication, although providers raised concerns that some patients might not log in. Most providers were less comfortable with upgraded VUS reclassifications being shared through a GHP. They felt these needed to be communicated by a genetic counselor and felt a message (eg, “please call your genetic provider”) was a more appropriate method for notification through a GHP.

#### Qualitative Patient Interviews

Patients (n=10) were aged 29 to 69 years and included 2 men, 2 patients with less than a college degree, 1 Black patient, and patients with a range of test results (ie, positive, VUS, negative, and results pending). Most participants (9/10, 90%) thought that a GHP could be useful in referencing immediate results, concise information, and accessing reports and documents. Suggestions from participants included the addition of content and documentation (ie, results, recommendation letter, and family history); information about individual risk compared with the general population; ability to track screening and medical management; and updates in the field. Most participants (8/10, 80%) supported receiving updated test results through a GHP. Participants also felt that a GHP could help support communication with relatives. They also suggested that a GHP could be more useful if it provided tailored educational resources, more billing and security information, a medical history summary, opportunities to connect with other patients, and if it were designed to be user friendly.

### Step 3: Developing the MyCancerGene Intervention

#### Patient-Facing Content

**Figure 1:**
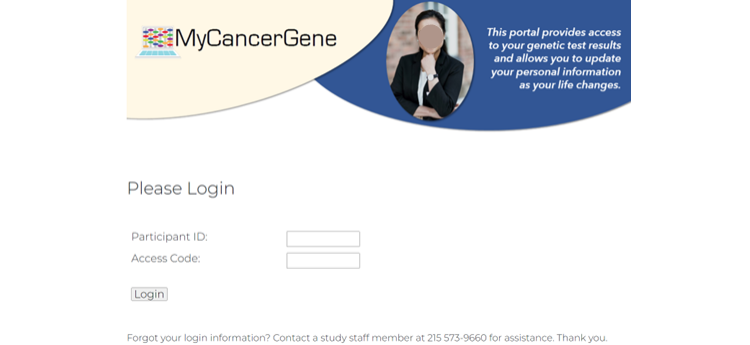
Log-in page.

**Figure 2:**
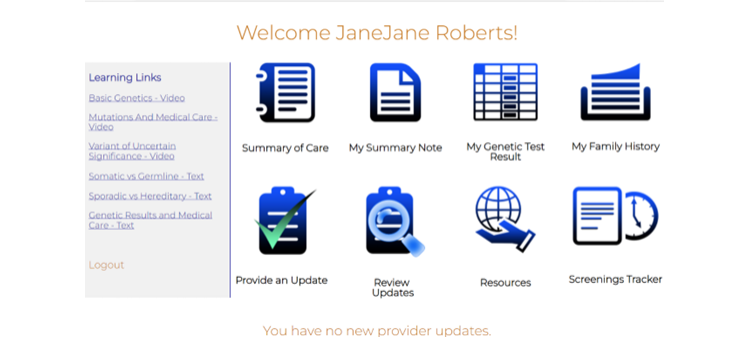
Landing page.

**Figure 3:**
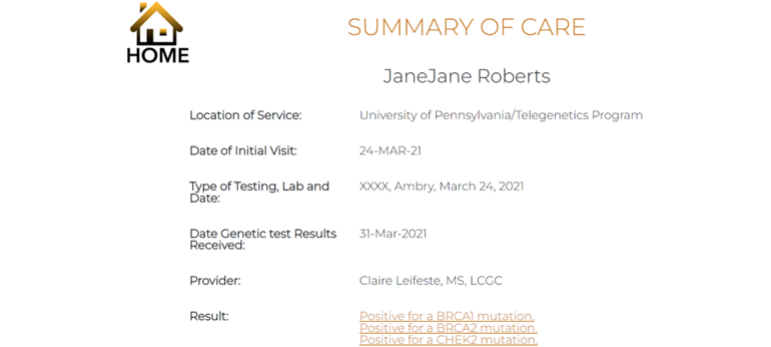
Summary of Care page.

**Figure 4:**
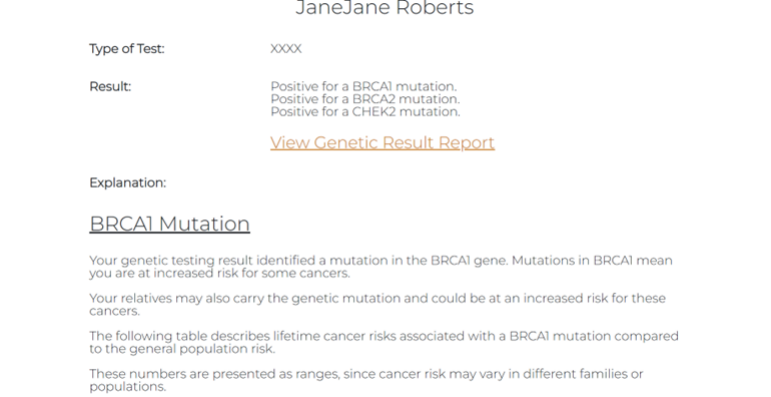
My Genetic Test Results page.

**Figure 5:**
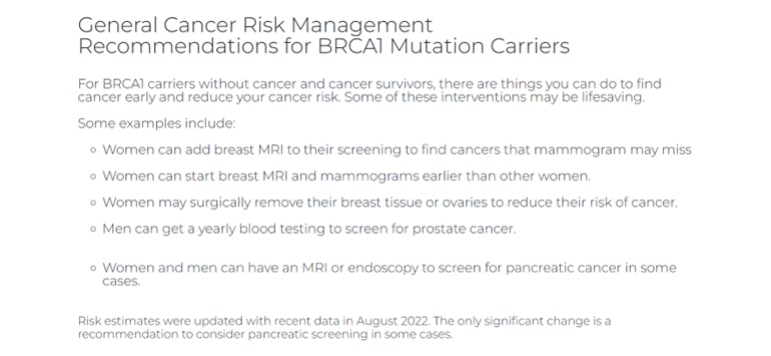
My Genetic Test Results page (continued).

**Figure 6:**
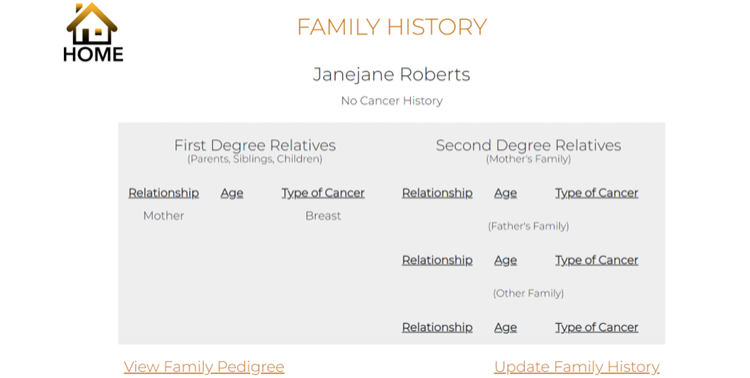
My Family History page.

Components of our GHP (ie MyCancerGene) were informed by our patient and genetic provider interviews as detailed in Table 7. Components included (1) the Log-in and Landing Page (Figures 1 and 2), (2) Summary of Care page (Figure 3), (3) My Genetic Test Results page (Figures 4 and 5), (4) My Family History page (Figure 6), (5) Provide an Update page, (6) Review Updates page, (7) Resources page, and (8) Screenings Tracker.

**Table 7:** Components of the MyCancerGene genetic health portal^a^.

Component	Description	Patient endorsement	Genetic provider endorsement
Landing Page	Includes 8 icons for intervention components and a list of learning links in the left side bar. There is a summary statement regarding any new updates and a reminder to provide an update if there is new family information.	“First page shouldn’t include any medical information. Users should be able to click and navigate to what they want to see and when.”	—^b^
Summary of Care	Includes location and date of service, testing laboratory, date and provider, test result statement (eg, positive for a BRCA1 mutation) and a link to the result page	“Would make things easier to recall when it is organized like this.”	—
My Genetic Test Results	Includes type of test, test result statement, and a PDF version to the test report. There is a patient-centered simple explanation or summary of the results, a table of lifetime risks associated, and general medical recommendations for positive results	“Would be likely to refer to this when sharing results, especially by phone.” “It’s like having an electronic file cabinet!”	“Could be an easier way for patients to share results with family and other providers.”
My Family History	Includes family history obtained at the medical visit and updated by the patient in the portal. There is a link to the PDF document of the last provider-generated pedigree and a link to update family history	“Super useful section. New doctor always asks for this.” “Big improvement than when [patient] had to fill it out by hand.”	“Some patients might not recall which relatives’ history they have or haven’t shared. Being able to view to verify before they reach out would be helpful.”
Provide an Update	Includes icons to provide a family update (family history or genetic testing in relatives), personal update (medical history of genetic testing), and other updates (contact information and other information)	“Feels there is a higher chance of ‘success’ in conveying updates or contact this way over a “standard” telephone approach. With a portal, there is a way to track who has been told what and when. With the phone/mail, who can say?”	“Many patients are under the impression we will call them when new testing or other information becomes available. This is often not feasible.”
Review Updates	Provides a chronological list of updates with dates, provider involved, and summary of the update	“[I] like this, but would probably be more inclined to update as needed when reminded.”	“Currently, certain updates are handled by letter. If a verification that update has been received is available, some updates may be easier this way.”
Resources	A list of 8 links for organizations that provide information regarding emotional and additional education resources. There is also a tool bar on the home screen with “Learning Links” including 3 videos and 3 text screens.	“[Would want] a section for recommendation links and resources”	—
Screenings Tracker	Includes the ability to enter the details of upcoming screening appointments including date, description, and comments. These are self-entered and self-monitored.	“Medical management recommendations for the patient specifically would be ideal...perhaps a way to track what screening/med. management had been done –a timeline.”	—

^a^There is a home icon on all component screens to return to the home screen. There is a “Have a question?” icon on every page.

^b^Not applicable.

#### Engagement Strategies

#### Overview

As endorsed by patients, reminders and engagement functions were designed to remind patients about the portal (Table 8). These include initial activation reminders to encourage patients to activate their MyCancerGene portal. We also designed general reminders sent every 6 months to remind patients to update their family or personal history and any new testing in the family. We also designed educational engagement reminders, which are short educational messages regarding cancer genetics or family risk and are sent every 6 months, alternating with general reminders (Table 8).

**Table 8:** MyCancerGene reminders.

Type	Frequency	Timing	Examples
Initiation reminder	4 reminders	On days 5, 9, 15, and 30	—^a^
General reminders (to update personal and family history and new testing in the family)	No end date; every 6 months	At 6, 12, and 18 months	Have you updated your family history: cancer status? Have you updated your family history: testing status?
Engagement reminders (including educational messages and reminders about genetic and familial risk)	No end date; every 6 months	At 3, 9, and 15 months	Have you shared your results with your relatives? Have you had your recommended screening?

^a^Not applicable.

#### Patient and Genetic Provider Updates

As outlined earlier, one of the main purposes of the MyCancerGene intervention is to provide a mechanism for patients to provide updates of their personal and family history to the genetic providers and for providers to send updates to the patients.

#### Patient-Driven Updates

MyCancerGene was designed so that patients can provide updates to their personal and family history or new testing in the family at any time on the Provide an Update page. They may also provide an update in response to general engagement messages sent every 6 months. All updates were designed to be reviewed by their genetic provider to determine if their update prompts a change in medical management or new genetic testing in them or their relatives. Given the integration challenges and concerns about burden on genetic providers, the research staff reviewed updates, drafted responses when appropriate, and obtained a final approved response from the genetic provider. Genetic provider responses to updates were then sent to patients through the MyCancerGene portal. It is intended that this could be better automated in the future after efficacy is established.

#### Genetic Provider–Driven Updates

MyCancerGene was designed for 2 specific genetic provider updates. First, to provide updates in VUS classifications (eg, VUS reclassification to benign or VUS reclassification to pathogenic or likely pathogenic). When genetic providers received a laboratory update on a patient enrolled in the MyCancerGene portal, they contacted the research team with the update. The genetic provider and research team created updated content for the Summary of Care, My Summary Note, and My Genetic Test Results pages. These changes were then programmed by the Clinical Research Computing Unit and, when completed, would generate a message to be sent to the patient. For example, “Your Variant of Uncertain Significance Result has been reclassified. To view the details of your reclassification now, please log onto your MyCancerGene portal.” This message provided log-in information and the option to speak with a provider to receive the updated information. For example, “If you would rather speak with your Genetic Counselor to discuss this update, please contact a member of the research study team who will help you set up a telephone call.”

In addition to patient-specific updates to results, the MyCancerGene intervention was designed to permit other updates based on changes in the field that might apply to a group of individuals. Examples include new screening recommendations for patients with a specific genetic mutation (eg, new screening recommendations for *PALB2* carriers), changes to risk estimates for particular genes, or new genetic testing options for patients with specific personal or family history. Thus, MyCancerGene was designed to allow edits to all patient-facing content to allow for these updates and a mechanism to message patients that updates had been made. In practice, updates were classified as “informational,” with a message notifying patients that there were changes made to their genetic information but that it likely did not change their current care. In contrast, other updates were classified as “potentially actionable,” and messages suggested that they access MyCancerGene to review the updates and contact their genetic provider if they had questions.

#### Genetic Provider Interface and Functionality

At the time of development, there was not an easy pathway for this intervention (ie, MyCancerGene) to integrate with the existing EHR. Furthermore, at the time of development, the use of MyPennMedicine, the institutional patient portal, was relatively limited and for messaging only. Given that the intervention could not be integrated with the EHR at the time of development, and genetic provider concerns about the burden of operating in 2 separate systems, we elected to not develop a provider-facing functionality. Rather, case report forms for research staff to enter messages and submit content to programmers were used. We planned that if the intervention had proven efficacy (eg, after a randomized trial), an integrated provider-facing interface would be developed. For the randomized trial, we planned that research staff would act as facilitators to populate the genetic information in MyCancerGene (eg, testing performed, copies of reports, and letters). In addition, to reduce human error, a second verification step was included to ensure accuracy (eg, a study genetic counselor or second research staff member verifies that there is accurate information in MyCancerGene before participant access). It was expected that these steps would be reduced over time as systems permitted greater integration or at the time of wider dissemination, after efficacy of the intervention had been proven.

#### Programming and Technology Specifications

The MyCancerGene participant portal is based on Oracle database tools that reference tables, functions, and procedures in an Oracle database. The participant is provided with a URL link that they enter into a standard web browser. The link invokes an Oracle REST Services call that connects to the appropriate database and schema to call a stored procedure that implements the log-in process. The participant is provided credentials by the MyCancerGene staff to complete the log-in. The log-in procedure passes the credentials to a registration procedure that checks the credentials and creates an active session if the log-in is valid. The active session includes an expiration time for security purposes. Once a session is established, a page-rendering procedure is invoked to read content from database tables and dynamically generate HTML code for display on the browser. All content for the portal is maintained in tables of the database. Study research staff use a separate application that was built using Oracle ApEX to enter participant specific information into Oracle tables that is used by the portal procedures to build the web screens. The page-rendering procedure handles page navigation as well as screen display. A log-out option on the main portal screen is used to terminate the session.

### Step 4: User Testing and Refinement of the Intervention

#### Patient User Testing

Participants for the first 8 user-testing interviews were from 4 of the COGENT sites. They were all female, were aged 39 to 57 years, included 1 Hispanic and 1 Black patient, and included 3 participants with less than a college degree and patients with a range of results (ie, 5 negative, 1 positive, and 2 VUS results). Most participants reported the content to be both what they expected and useful. Key feedback from initial interviews included adding a landing page to be seen before the summary of care, reducing text, and suggestions for descriptive text, colors, and fonts. Participants were evenly divided on presenting family history information as a list versus as a pedigree. They also reported that general reminders and notifications when MyCancerGene information was updated would be important to increasing use of the intervention. Other recommendations included the addition of educational resources, details on care providers, and dates of visits and other events.

Participants for the second set of patient user–testing interviews (n=20) were identified from recent clinical encounters. They were aged 29 to 72 years, including 5 men and 1 participant with less than a college degree and with a range of results (ie, 3 negative, 10 positive, and 7 VUS results). Participants had limited feedback for most content, with most suggestions for the Landing Page, My Family History page, messages about updates, and general suggestions to increase use. Suggestions for the Landing Page included clarifying the organizational affiliation and changes to design, fonts, and images. Suggestions on the Family History page included the option to enter relatives and their test results, inclusion of third and extended generations, and the option to view a pedigree. Other general suggestions included replace pie charts with numerical risks, include a glossary and define terms, clarify the type of update and a link to the portal in messages, add educational resources, make MyCancerGene accessible on mobile devices, integrate MyCancerGene into the existing medical record and patient portal, and provide reminders about availability of MyCancerGene. Some participants expressed concerns about privacy and security and provided recommendations to reassure them (eg, use a secure site and authentication strategies).

Participants in the second set of interviews also reviewed several options for messaging for updates to their test results. In contrast to genetic provider opinions as described subsequently, they strongly preferred the option to receive a message and directly access their updates in MyCancerGene, with the option to speak with a genetic provider of their choice, as opposed to options which alerted them of an update but then required that they speak with a genetic provider first before having access to their update (ie, provider disclosed).

#### Genetic Provider User Testing

Of the 25 genetic counselors who completed user-testing interviews, 88% (22/25) were female, 32% (8/25) were affiliated with the University of Pennsylvania, and 68% (17/25) were from external practices from 5 states (Pennsylvania, New Jersey, Texas, California, and Florida). Genetic providers reported that the benefits to patients included providing a centralized area for information and a tool to communicate updates (Table 9). Providers also identified benefits for themselves, including optimizing communication and saving their time. They also reported potential challenges, including creating extra work and potentially leading to inefficiencies (Table 9).

Genetic providers also had recommendations for changes to the Summary of Care page, Landing Page, My Family History page, and VUS reclassification page, which included simplifying text, changes to graphics and pictures, adding screening recommendations or care plans, changes to identify relatives on the family history page, and accounting for multiple VUS results. Providers reported variable comfort with different types of updates being provided through MyCancerGene (Table 10). They expressed high comfort with updates to general testing information, reminders, and downgrades to VUS results. However providers varied in their comfort with upgraded VUS results being provided through MyCancerGene. Some were comfortable providing patients with the option to directly access their updates in the portal, with the option to speak with a provider instead if they preferred. Others felt that changes in VUS classification (ie, particularly upgrades) could be sent as a message, which would alert patients of the update, but they felt that patients should be required to speak with a provider first before accessing their update in MyCancerGene.

**Table 9:** Key findings from genetic provider stakeholder interviews and user testing (n=25).

Questions	Most common themes: n (%)	Examples
When thinking about your patients, how do you think this patient portal (MyCancerGene) would benefit them?	Centralized information: 13 (59) Clearer updates: 8 (36)	“All documentation would be easy to locate. Family history and VUSa updates which are typically cumbersome would be streamlined.”
What types of challenges could MyCancerGene alleviate for genetic counselors?	Centralized information: 8 (33) More efficient communication: 8 (33) Saves provider’s time: 7 (29)	“Could increase efficiency (less back and forth phone calls/sending letters that may or may not get to where they need to, etc...)” “Could end up saving as much as 30 minutes with each interaction since, instead of having to scavenge through chart notes, all the pertinent points are highlighted for each patient.”
What types of challenges could MyCancerGene create for genetic counselors?	Extra work: 15 (63) Inefficient communication: 5 (20.8)	“More work (double the work if not integrated with the EMRb). Also potential for more work if patients have an easier means of communication.”

^a^VUS: variants of uncertain significance.

^b^EMR: electronic medical record.

**Table 10:** Genetic provider comfort with different types of provider updates.

Comfort with different types of updates	Most common themes: n (%)	Examples
General testing information	Comfortable: 23 (92)	“Comfortable. Thinks this is so much better than the ‘call us to follow up to see if there are any updates.’”
Reminders	Comfortable: 24 (96)	“Comfortable - and patients would appreciate this.”
Downgraded VUS^a^	Comfortable: 22 (88)	“Comfortable with confirmation.”
Upgraded VUS	Comfortable: 11 (44) Not comfortable: 11 (44)	“Comfortable as long as follow-up with GCb is not just suggested but strongly encouraged if not mandatory.” “Not comfortable; prefers to call patients directly.”

^a^VUS: variants of uncertain significance.

^b^GC: genetic counselor.

### Step 5: Usability Testing and Final Modifications to the Intervention

Participants for usability testing (n=8) were White women who were aged 24 to 70 years, with a range of education levels (ie, high school only to graduate level). Participants had a range of genetic test results (ie, 6 positive, 1 VUS, and 1 negative). Overall, many participants reported that icons and features were easy to understand, useful, and what they expected. Participants reported greatest interest in the Genetic Test Results and Family History pages and additional resources. While they reported the summary letter was useful to have, several mentioned that they may not use it. Some were not clear what it was, even though they should have received a copy after their genetic counseling disclosure visit. Most found the Summary of Care and Summary of Updates pages useful, and most felt the Review Updates page was easy to understand. There were several typos or spacing recommendations to make text easier to understand, and these were generally adopted. Recommendations for changes to fonts, pictures, or colors were made when recommendations were not conflicting or were mentioned by more than 1 participant. Key recommendations included making pages available in a printable format, summaries under the videos, a note that if something does not appear accurate (eg, personal or family history) to go to share an update, and the ability to provide more notes on the family history page (eg, dates of diagnosis). Another recommendation was to add the My Screenings page, which had been a previous recommendation that was not included due to the challenge of keeping it updated with the electronic medical record when MyCancerGene was not yet integrated. However, given the repeated patient feedback, this page was created in the final version so that participants could self-track screening and data. Other recommendations that were considered but were not possible at the time included providing access to relatives, providing access to genetic providers, synchronizing a screenings tracker with their calendar, making the intervention available within their existing portal, and creating a repository where patients could store articles and websites they found personally useful. These were not included because they were either suggested by a single participant or were technically challenging at the time, but they were recorded as potential future considerations.

## Discussion

### Principal Findings

With the advent of MGPT in clinical genetic testing, an increasing number of patients are left at a risk of misunderstanding, uncertainty, and evolving interpretations and recommendations after receipt of cancer genetic test results [6,8,71]. Thus, longitudinal follow-up to update risk estimates and recommendations for positive results, update VUS results, and update family history based on changing circumstances are needed. Yet, cancer genetic testing is often a one-time encounter with a genetic counselor, as opposed to an ongoing relationship with continuity of care. This leaves the responsibility for follow-up, updates, and longitudinal care to patients, who can contact their genetic provider as needed, or to their other health care providers, who may not be equipped to provide genetic-specific updates. To address this clinically significant gap in cancer genetic care, we developed a patient-centered longitudinal digital GHP to support patients with longitudinal care after receipt of their cancer genetic test results. MyCancerGene was directly informed by the feedback from patients and genetic providers directing both the content and functionality of the intervention. The initial prototype was then refined through extensive user and usability testing with patients who had received a range of genetic test results.

Patient and genetic provider interviews identified high interest in a patient-centered longitudinal digital GHP to support longitudinal care. Over 90% of patients with positive results and 75% of participants with a VUS or uninformative negative results reported an interest in a patient-centered digital GHP. The primary advantages of this type of tool, according to patients, included increased accessibility, convenience, and efficiency of accessing their genetic test reports and other documentation from the genetic visits, keeping genetic information organized, and increasing and maintaining patient understanding through easy-to-understand materials and educational resources. Patients also felt that such a digital health tool could help with communication and sharing of materials with relatives or other health care providers. Patients were also in favor of receiving electronic updates through a patient-centered digital GHP and highlighted that the intervention would need to address privacy concerns and be easy to use. Similar to patients, genetic providers reported that a patient-centered digital GHP could help patients share information with relatives and other health care providers and help patients to update the genetics team about new health information (ie, personal or family history or new test results in the family). Patients and genetic providers also felt the portal may help provide a mechanism to update patients with new information about genetic results. While many providers endorsed this tool as a place to electronically store documents for patients, some noted that this could be redundant to what is already provided in the electronic health portal, and the added value was unclear.

A key component of this formative work was to determine, from the patient and genetic provider standpoint, what content and functions this patient-centered digital GHP (ie, MyCancerGene) should include. Patient and provider input identified 8 key components of the tool and most were endorsed by both patients and providers as useful for longitudinal care. These included the following pages: Landing Page, Summary of Care, My Genetic Test Results, My Family History, Provide an Update, Review an Update, Resources, and a Screenings Tracker. Patients and providers also addressed several key functions, including the ability to download and print materials and the inclusion of reminders and engagement functions. The iterative user and usability testing helped inform changes to increase ease of access by making the layout and design more intuitive, changes to content to increase understanding, the inclusion of a glossary with defined terms, the addition of educational resources, and changes to pictures and colors to help with patient understanding and overall experience. Integrating MyCancerGene into other health portals, providing access to relatives, and synchronizing screening trackers with personal calendars were unable to be incorporated at the time but were identified as future content or functions that could be helpful.

While most patients and genetic providers endorsed advantages and benefits of a patient-centered digital GHP, some identified potential challenges. Some participants had privacy or security concerns, and a few anticipated that reviewing past or updated information regarding genetic risk electronically could be upsetting or distressing. A few also commented that MyCancerGene would be yet another health portal that could create additional log-in credentials, which can be hard to recall. Patient and provider comfort levels also varied with the return of updated VUS results in MyCancerGene. While patients were comfortable with updates (ie, even VUS reclassification upgrades or clinically significant results), genetic providers had mixed feelings on the appropriateness of sharing upgraded VUS results through MyCancerGene. After sharing the overwhelming support among patients to make a choice for themselves if they are ready for updated information about their results, genetic providers were more open to this option, although some still had reservations. This will be an important outcome to evaluate in future longitudinal studies given the variable opinions from patients and genetic providers.

Another primary challenge identified by both patients and genetic providers is the strong desire to have such a genetic portal integrated with the existing EHR. Technological challenges in integrating the Oracle based system with the existing EHR and long queues and prioritization for already existing EHR modifications were barriers to developing MyCancerGene initially as an integrated component of the EHR. Furthermore, it was felt that establishing efficacy would be important to determining prioritization, value, and investment in future integration. Another concern was the availability of a variety of different EHR platforms available and institutions might differ in their choice of platform used, which could limit future implementation. For these reasons, the intervention was built as a proof of concept for a randomized trial with plans to engage an IT integration committee throughout the trial to consider how MyCancerGene could be integrated with the EHR in the future. Of note, the genetic provider–facing component of MyCancerGene would need to be developed and tested at the time of future integration.

### Limitations

Some limitations to our formative research and development are acknowledged. The hypothetical query may not have aligned with future use and benefits. Thus, a randomized trial of the intervention compared to usual care is planned and may better define real-world benefits and outcomes. As noted earlier, some desired components and functions could not be included, and this may limit the benefits, although these could be developed for future versions of MyCancerGene. In addition, while we attempted to purposefully identify sociodemographically diverse patients, some declined participation, and given funding timelines, the user-testing evaluations were more limited in diversity and could have benefited from a more diverse patient population. Recruiting from both academic and community settings in the future randomized controlled trial is planned to assess the real-world benefits of a GHP and to reduce the possibility of contributing or exacerbating existing digital disparities [5,44,72].

### Randomized Trial

The efficacy of MyCancerGene is being evaluated in a randomized study (NCT04774445) and compared to usual care following the receipt of genetic testing results in real-world clinical patients. In this ongoing study, we hypothesize that the intervention will be associated with short-term and longitudinal increases in knowledge, decreases in distress, increases in communication with relatives and health care providers, and increases in adoption of risk-reducing health behaviors.

### Conclusions

The MyCancerGene digital GHP was developed with extensive feedback from patients and genetic providers and may be a useful digital health tool to enhance longitudinal patient understanding of and affective and behavioral responses to genetic testing, particularly in the era of evolving evidence and risk information. Evaluation of MyCancerGene in a randomized trial in real-world clinical settings will determine the real-world uptake and clinical risks and benefits of this portal, which will ultimately contribute to the integration and promise of personalized genetic medicine.

## Abbreviations

COGENT: Communication of Genetic Test Results by Telephone
EHR: electronic health record
GHP: genetic health portal
MGPT: multigene panel testing
OR: odds ratio
VUS: variants of uncertain significance

## Data Availability

The datasets generated and analyzed during this study are available from the corresponding author on reasonable request.
